# Aloperine inhibits the progression of non-small-cell lung cancer through the PI3K/Akt signaling pathway

**DOI:** 10.1186/s12935-021-02361-5

**Published:** 2021-12-11

**Authors:** Fujuan Liu, Tao Liu, Haiying Li

**Affiliations:** 1 Department of Pharmacy, Linyi Fourth People’s Hospital, No. 121, Qianshi Ave., Linyi, 276005 Shandong China; 2 Department of Pediatrics, Linyi Fourth People’s Hospital, No. 121, Qianshi Ave., Linyi, 276005 Shandong China; 3 Department of Ultrasound, Qilu Hospital of Shandong University, No. 107, Wenhuaxi Rd., Jinan, 250012 Shandong China

**Keywords:** Aloperine, NSCLC, PI3K/Akt pathway, Tumor progression

## Abstract

**Background:**

Lung cancer has become the leading cause of cancer-related death worldwide and non‐small‐cell lung cancer (NSCLC) accounts for approximately 85% of cases. Aloperine (ALO), an alkaloid active natural component from *S. alopecuroide*, has been found to exhibit anti-inflammatory, anti-tumor and anti-viral activity. However, Whether ALO exerts anti-tumor function on NSCLC remains poorly understood, and the underlying mechanisms remain unknown.

**Methods:**

The CCK-8, colony formation, cell apoptosis with flow cytometry, wound healing and transwell cell invasion assays, were used to analyze the tumor progression of H1299 and A549 cells treated with ALO in vitro, and the xenograft model was constructed to assess the effect of ALO in vivo. The expression of protein was detected by Western blotting.

**Results:**

ALO suppressed the cell proliferation, self-renewal, migration and invasion, induced apoptosis in A549 and H1299 cell. Furthermore, ALO significantly enhanced the level of cytochrome c in cytosol, and resulted in the dramatical increased levels of the cleaved caspase-3, caspased-9 and PARP. ALO also inhibited the expression of MMP-2 and MMP-9. Additionally, ALO also reduced p-AKT and p-mTOR to attenuate the PI3K/AKT signaling pathway.

**Conclusion:**

This study unveils a rationale for ALO through PI3K/Akt signaling pathway affecting the cell progression such as cell growth, apoptosis and invasion, and ALO acts as a potential chemotherapeutic agent for NSCLC.

## Introduction

Lung cancer (LC) is the most common cause of cancer death worldwide [1]. Non‐small‐cell lung cancer (NSCLC) is the most histological subtypes of LC, accounting for 85% of the total number of LC [2]. Currently, surgery, radiotherapy and chemotherapy are the main therapies for lung cancer. However, the treatment effects are not effective and have adverse effects. Thus, it is necessary to develop novel treatment strategies that effectively treat NSCLC. Natural products can exhibit many beneficial effects on human health and have lower side effects in treatment cancers [3], there is growing concern about plant-derived drugs as an alternative to cancer therapy. Identifying of new compounds among natural sources with chemotherapeutic properties is urgent.

Aloperine (ALO) is a quinolizidine alkaloid drug that is extracted from *S. alopecuroides*, which is a traditional Chinese medicine [4]. ALO has been shown to exhibit anti-inflammatory, anti-allergenic and antiviral activity [5–8]. More importantly, emerging evidences indicates that ALO exerts anti-tumor functions on multi-tumors such as hepatocellular carcinoma [9], osteosarcoma [10], colon cancer [11], breast cancer [12]. However, whether ALO exerts anti-tumor activities on NSCLC is unknown, and the underlying mechanisms have not been defined.

Apoptosis is a programmed form of cell death and represents the major mechanism of cell death in cancer therapies [13, 14]. Apoptosis can be triggered through either extrinsic or intrinsic pathways. Both pathways converge to activate the effector apoptotic caspases (i.e. caspases-3, -6 and -7) resulting in apoptotic cell death [15]. The intrinsic pathway is primarily regulated by the Bcl-2 family. Bcl-2 and Bax are the two main proteins that play a key role of pro-survival and pro-apoptotic functions, respectively. Upon activated, Bax forms multispanning monomers that oligomerize to permeabilize mitochondrial membranes, leading to the proteins normally retained in the intermembrane space spread into the cytosol^16^. Additionally, Matrix metalloproteinases (MMP) including MMP-2 and MMP-9 play a major role in tumor invasion by degrading the extracellular matrix [17]. It has been reported that MMP-2 and MMP-9 are essential in in NSCLC invasion and metastasis [18, 19].

It is well known that the phosphatidylinosito-3-kinase/protein kinase B (PI3K/Akt) signaling pathway plays vital role in the growth and survival of cancer cells [20, 21]. Notably, abnormal Akt activation is a poor prognostic factor for all stages of NSCLC [22]. Moreover, activated AKT protects NSCLC cells from chemotherapy and radiation-induced apoptosis [23, 24]. Thus, inhibition of the PI3K/Akt signaling pathway is an attractive therapeutic strategy in NSCLC.

Here, we found that ALO suppressed cell proliferation and induced apoptosis in via promoting intrinsic apoptotic pathway in both A549 and H1299 NSCLC cells. Besides, ALO inhibited the invasion in A549 and H1299 cells through downregulated MMP-2 and MMP-9 expression. More importantly, ALO attenuated PI3K/Akt signaling pathway, which is involved in tumor cell proliferation, metastasis and apoptosis.

## Materials and methods

### Cell culture and reagents

The human NSCLC cell lines H1299 and A549 were purchased from ATCC and cultured in DMEM (Thermo Scientific) supplemented with 10% fetal bovine serum (Thermo Scientific) and 1% penicillin and streptomycin at 37 °C in a humidified atmosphere containing 5% CO_2_. ALO and SC79 (p-AKT agonist) [25] were purchased from Selleckchem and dissolved in Ethanol.

### Cell viability assay

H1299 and A549 cells were counted and seeded at 5 × 10^3^cells/ well into each 96-well overnight for cell adhesion and treated with fresh DMEM containing ethanol or fresh DMEM with different doses (0, 0.2, 0.4, 0.8, and 1 mM) of ALO for indicated time points. Then, 10ul of CCK-8 solution (MedChemExpress) was added to each well and incubated for another 1 h at 37 °C. Measure the absorbance at 450 nm and 650 nm using a microplate reader (Thermo Scientific).

### Colony formation assay

H1299 and A549 (1 × 10^3^cells per well) were resuspended and plated in 6-well plates. After 24 h, cells were treated with the indicated dose of ALO for 15 days. Then, cells were fixed with 4% paraformaldehyde and stained with 0.1% crystal violet for 1 h. Images were obtained by using a digital camera at 4× magnification.

### Cell apoptosis analysis 

H1299 and A549 (4 × 10^5^/well) were seeded in 6-well plates for 24 h and then treated with ALO for 24 h. Cells were harvested and washed with cold PBS, the cell pellets were then resuspended with binding buffer (400 μl) and stained with Annexin V-FITC and PI (Vazyme Biotech) according to the protocols. The apoptotic cells ware analyzed by using FACS Calibur flow cytometer (BD Biosciences).

### Wound healing assay

H1299 and A549 (2 × 10^6^/well) were seeded in 6-well plates. After cells converged almost 100%, scratch was made in the plate using a P200 pipette tip. The wound was generated and then washed the cells with PBS. Added medium with or without ALO to the cells and incubated for another 24 h. The scratched area was photographed with an Olympus microscope at 0 h and 24 h with 4× magnification, respectively.

### Trans well cell invasion assay

Trans well chambers (8-μm pore size Corning) were used to assay the invasive ability of the H1299 and A549. The cells (4 × 10^4^ cells/200 μl) were suspended in the FBS-free DMEM and were placed in the Matrigel (BD Biosciences) pre-coated upper chamber, following which 500 μl of complete DMEM containing 10% FBS was added to the lower chamber. Following treated with or without ALO for 24 h, the invaded cells were fixed and then stained with 0.5% Crystal violet solution for 1 h. The stained cells were observed under a light microscope at 10× magnification, and 5 fields per chamber (insert) were observed for counting invaded cell numbers.

### Western blot

H1299 and A549 cells treated with ALO for 24 h were harvested and washed with cold PBS. After that, cells were lysed with RIPA Reagent (Beyotime Biotechnology) supplemented with a protease inhibitor cocktail (Sigma) and incubated on ice for 30 min. Then, the cells were centrifuged at 12,000×*g* for 20 min at 4 °C. The proteins were quantified using the BCA Protein assay kit (Thermo Scientific). Equal amounts of extracts (50ug) were separated by SDS-PAGE and then were transferred onto nitrocellulose membranes for immunoblot analysis. The cytoplasmic protein was extract by PARIS™ kit protein and RNA isolation system (Thermo Scientific) for cytochrome c analysis. The following antibodies were used at a dilution of 1:1000 unless otherwise stated: anti-β-actin (Santa Cruz, sc-8432, 1:2000), anti-Cleaved Caspase-3 (Cell Signaling Technology, 9664), anti-Cleaved Caspase-9 (Cell Signaling Technology, 9509), anti-Cleaved PARP (Cell Signaling Technology, 94,885), anti-Cytochorome c (Cell Signaling Technology, 4850), anti-MMP-2 (Cell Signaling Technology, 40,994), anti-MMP-9 (Cell Signaling Technology, 13,667), anti-AKT (Cell Signaling Technology, 4691), anti-p-AKT (Cell Signaling Technology, 4060), anti-mTOR (Cell Signaling Technology, 2983) and anti-p-mTOR (Cell Signaling Technology, 5536).

### Xenograft assay

All animal procedures were performed in accordance with protocols of the Care and Use of Laboratory Animals approved by the Animal Ethics Committee of Linyi Fourth People’s Hospital. The procedure for the xenograft assay, H1299 cells (2 × 10^6/^100 μl) were subcutaneously injected into the right flank of 4-weeks-old BALB/C nude mice (SiPeiFu Experimental Animal). When the tumor volume reached 100 mm^3^, the animals were randomly divided into Mock group and ALO treatment group, 10 mice in each group. The ALO treatment group was given intraperitoneal injection of ALO at a dose of 30 mg/kg and the Mock group was given intraperitoneal injection of normal saline at equal volume for 15 consecutive days, and all the mice were sacrificed 28 days later. The euthanasia method was followed as using one hand to hold the animal and tilt the animal slightly downward so that its head lower than its abdomen, injects sodium pentobarbital (200 mg/kg) into the caudal left quadrant. Tumor volumes were measured every day and calculated using the formula: volume = 0.5 × length × width^2^.

### Statistical analysis

All data are expressed as the mean ± S.D. of three or more experiments and analyzed by Graphpad Prism 5.0 software (Graphpad software Inc., CA, USA). Statistical significance was determined with the two-tailed Student’s t-test. The significance was determined: *P < 0.05, #P < 0.05 and &P < 0.05, with NS indicating no significance.

## Results

### ALO suppresses cell proliferation and self-renewal

**Fig. 1:**
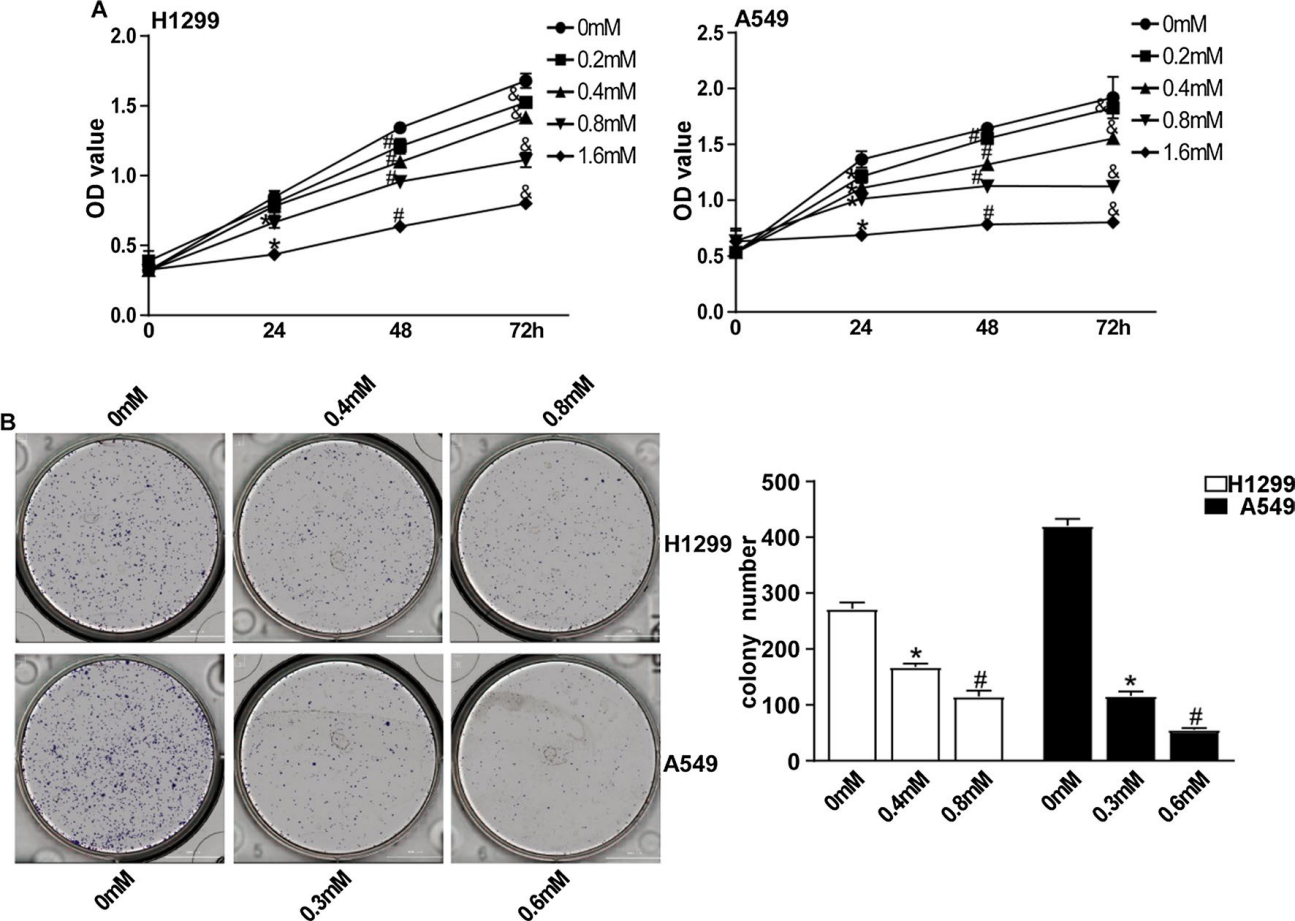
Inhibition effect of ALO on the proliferation and self-renewal of NSCLC cells. **A** H1299 and A549 cells were treated with ALO for 24, 48 and 72 h at 0.2, 0.4, 0.8 and 1.6 mM, respectively, cells proliferation was detected by CCK8 assay. *P < 0.05, the ALO group (0.2, 0.4, 0.8 and 1.6 mM) compared to the control group (0 mM, solvent ethanol) at 24 h; #P < 0.05, the ALO group (0.2, 0.4, 0.8 and 1.6 mM) compared to the control group (0 mM, solvent ethanol) at 48 h; &P < 0.05, the ALO group (0.2, 0.4, 0.8 and 1.6 mM) compared to the control group (0 mM, solvent ethanol) at 72 h. **B** H1299 and A549 cells were treated with the indicated dose of ALO, the colony formation viability was evaluated by clonogenic formation assay. *P < 0.05, compared to the control group (0 mM, solvent ethanol); #P < 0.05, compared to the ALO group (0.4 mM or 0.3 mM). Data are from one experiment representative of three experiments. Two-tailed Student’s test

To evaluate whether ALO treatment suppresses cell growth in NSCLC cells, H1299 and A549 cells were treated with different concentrations of ALO (0, 0.2, 0.4 0.8 and 1.6 mM) for 24, 48 and 72 h respectively. Then the cell viability was determined using CCK8 assay. ALO inhibited the proliferation of both H1299 and A549 cells in dose-dependent manners (Fig. 1A). Moreover, the 50% inhibitive concentration (IC50) values of ALO were calculated. The IC50 values in H1299 and A549 cells were around 0.8 mM and 0.6 mM after ALO treatment for 72 h, respectively. Therefore, we selected 0.4 mM and 0.8 mM for H1299 cells, 0.3 mM and 0.6 mM for A549 cells treatment in the following experiments. In addition, colony formation assay confirmed that the effect of ALO on cell self-renewal of both cell lines, compared to untreated cells, ALO treatment inhibited the colony formation capacities of both H1299 and A549 cells in a dose-dependent fashion (Fig. 1B). Therefore, the results indicate that ALO suppresses the proliferation and self-renewal in both A549 and H1299 NSCLC cells.

### ALO induces apoptosis in NSCLC cells via intrinsic apoptotic pathway

**Fig. 2:**
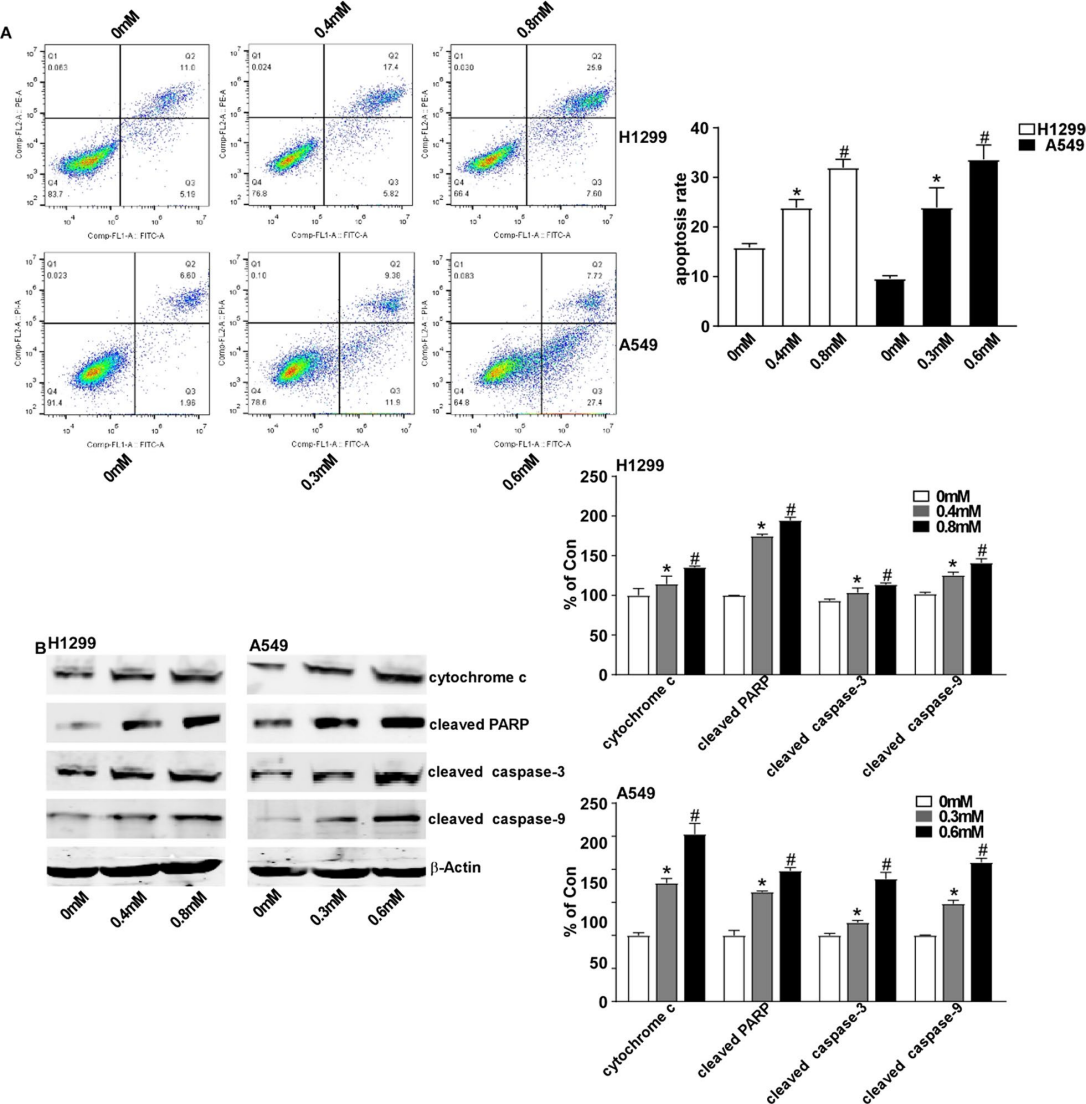
Induction effect of ALO on the apoptosis of NSCLC cells via intrinsic apoptotic pathway. **A** H1299 and A549 were treated with ALO for 24 h, and then were stained with Annexin V-FITC/PI, then analyzed by flow cytometry. Annexin V labeled with FITC used to identify early apoptotic cells, PI used to distinguish survival early cells from necrotic or late apoptotic cells. Apoptosis rate means the ratio of the amount of early and late apoptotic cells to the total amount of cells. *P < 0.05, compared to the control group (0 mM, solvent ethanol); #P < 0.05, compared to the ALO group (0.4 mM or 0.3 mM). **B** H1299 and A549 were treated with ALO for 24 h, and then cells were lysed with RIPA buffer. The cell lysates were analyzed by Western blotting with the indicated antibodies. *P < 0.05, compared to the control group (0 mM, solvent ethanol); #P < 0.05, compared to the ALO group (0.4 mM or 0.3 mM). Data are from one experiment representative of three independent experiments. Two-tailed Student’s test

To investigate whether ALO inhibits the cell viability via apoptosis induced in NSCLC cells. Apoptosis analysis was conducted in H1299 and A549 cells following ALO treatment for 24 h. The percentage of apoptotic cells both increased with a dose dependent fashion in H1299 and A549 cells (Fig. 2A). To explore the underlying mechanisms of ALO induced apoptosis in NSCLC cells, the expression levels of cytochrome c and the cleavage of caspase-3, caspased-9 and PARP were subsequently measured by Western blot, which are the key regulators of the intrinsic apoptotic pathway [26]. ALO treatment significantly enhanced the level of cytochrome c in cytosol, and resulted in the dramatical increased levels of the cleaved caspase-3, caspased-9 and PARP (Fig. 2B). These findings reveal that ALO induces apoptosis in NSCLC cells via intrinsic apoptotic pathway.

### ALO attenuates the migration and invasion in in NSCLC cells by downregulating MMPs

**Fig. 3:**
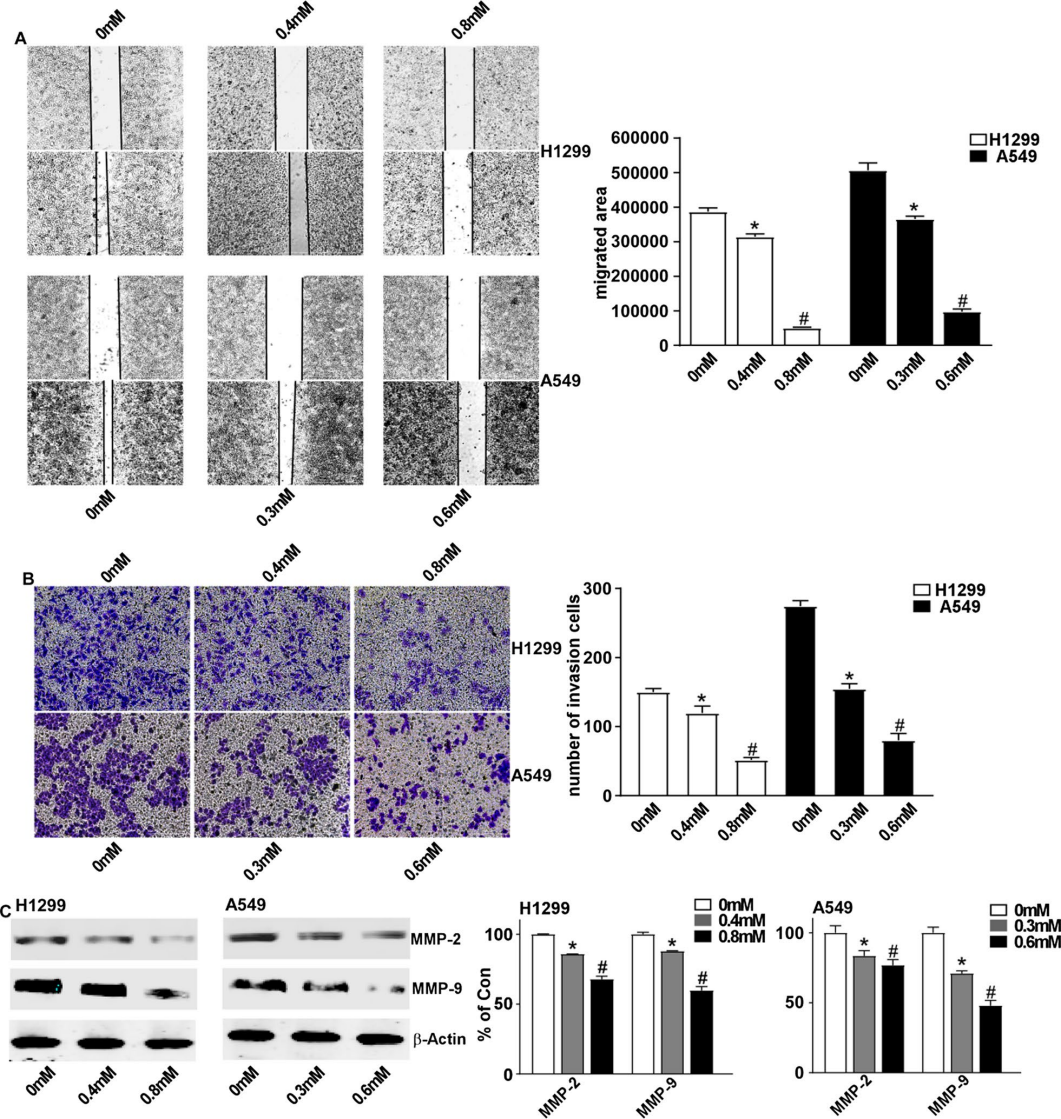
Suppression effect of ALO on the migration and invasion of NSCLC cells via MMPs. **A** H1299 and A549 were treated with ALO for 24 h, the cells migration was detected using wound healing assay. *P < 0.05, compared to the control group (0 mM, solvent ethanol); #P < 0.05, compared to the ALO group (0.4 mM or 0.3 mM). **B** H1299 and A549 cells were treated as in A, the cells invasion was detected by transwell assay. *P < 0.05, compared to the control group (0 mM, solvent ethanol); #P < 0.05, compared to the ALO group (0.4 mM or 0.3 mM). **C** H1299 and A549 cells were treated with indicated concentrations of ALO for 24 h, and then cells were lysed with RIPA buffer. The cell lysates were analyzed by Western blotting with the indicated antibodies. *P < 0.05, compared to the control group (0 mM, solvent ethanol); #P < 0.05, compared to the ALO group (0.4 mM or 0.3 mM). Data are from one experiment representative of three experiments. Two-tailed Student’s test

To explore whether ALO treatment also affects the migration and invasion abilities in NSCLC cells, we performed wound healing and transwell invasion assay. Wound healing assay showed that ALO treatment for 24 h significantly attenuated cell migration in H1299 and A549 cells in a dose dependent fashion (Fig. 3A). Further, transwell assay indicated that ALO treatment inhibited the invasion of NSCLC cells transit from the matrigel-coated membrane in a dose-dependent manner (Fig. 3B). The MMP-2 and MMP-9 play important roles in the invasion and metastasis of NSCLC cells. Therefore, we examined the levels of MMP-2 and MMP-9 after treating with ALO by western blot. The following treatment with ALO for 24 h, western blot analysis revealed that the levels of MMP-2 and MMP-9 were markedly reduced in both A549 and H1299 cells (Fig. 3C). These data indicates that ALO suppresses the migration and invasion of A549 and H1299 cells by downregulated of MMP-2 and MMP-9 expression.

### ALO inhibits PI3K/AKT/ mTOR signaling pathway

**Fig. 4:**
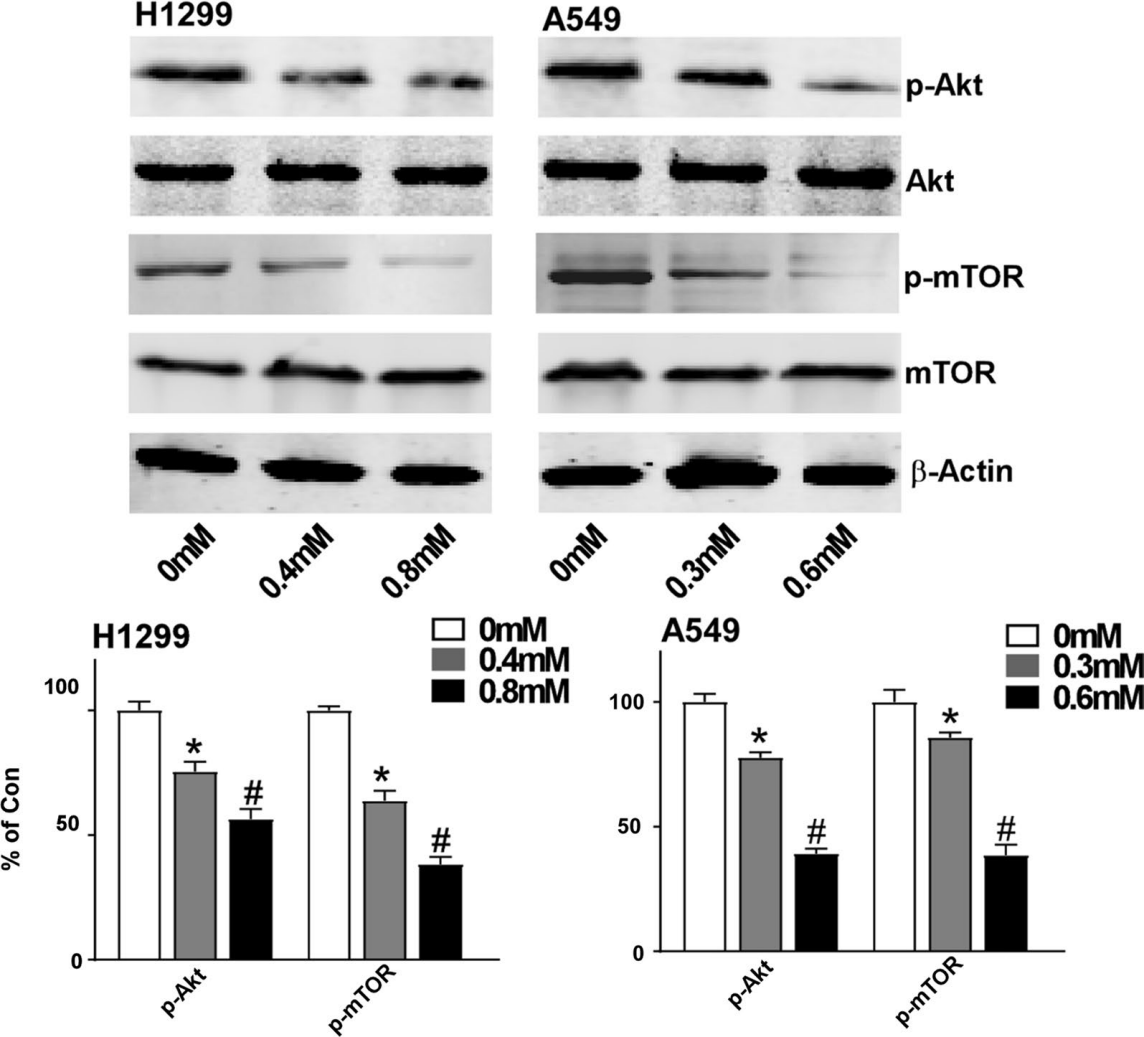
PI3K/AKT/ mTOR signaling pathway, the target of ALO. A549 and H1299 cells were treated with indicated concentrations of ALO for 24 h, and then cells were lysed with RIPA buffer. The cell lysates were analyzed by Western blotting with the indicated antibodies. *P < 0.05, compared to the control group (0 mM, solvent ethanol); #P < 0.05, compared to the ALO group (0.4 mM or 0.3 mM). Data are from one experiment representative of three experiments. Two-tailed Student’s test

PI3K/AKT/mammalian target of rapamycin (mTOR) pathway is one of the most frequently activated signaling pathways in NSCLC [27, 28], and is involved in cell apoptosis as well as invasion. To explore whether administration of ALO affects this pathway, H1299 and A549 cells were treated with or without different concentrations of ALO for 24 h, and the status p-Akt and p-mTOR were determined by western blot. The phosphorylation of Akt and mTOR were reduced after ALO treatment, however, the expression of Akt and mTOR were unchanged by ALO treatment (Fig. 4). The results suggest that ALO might induce apoptosis and invasion of NSCLC cells by inhibiting PI3K pathway.

### ALO influence cell proliferation and apoptosis through PI3K/AKT pathway

**Fig. 5:**
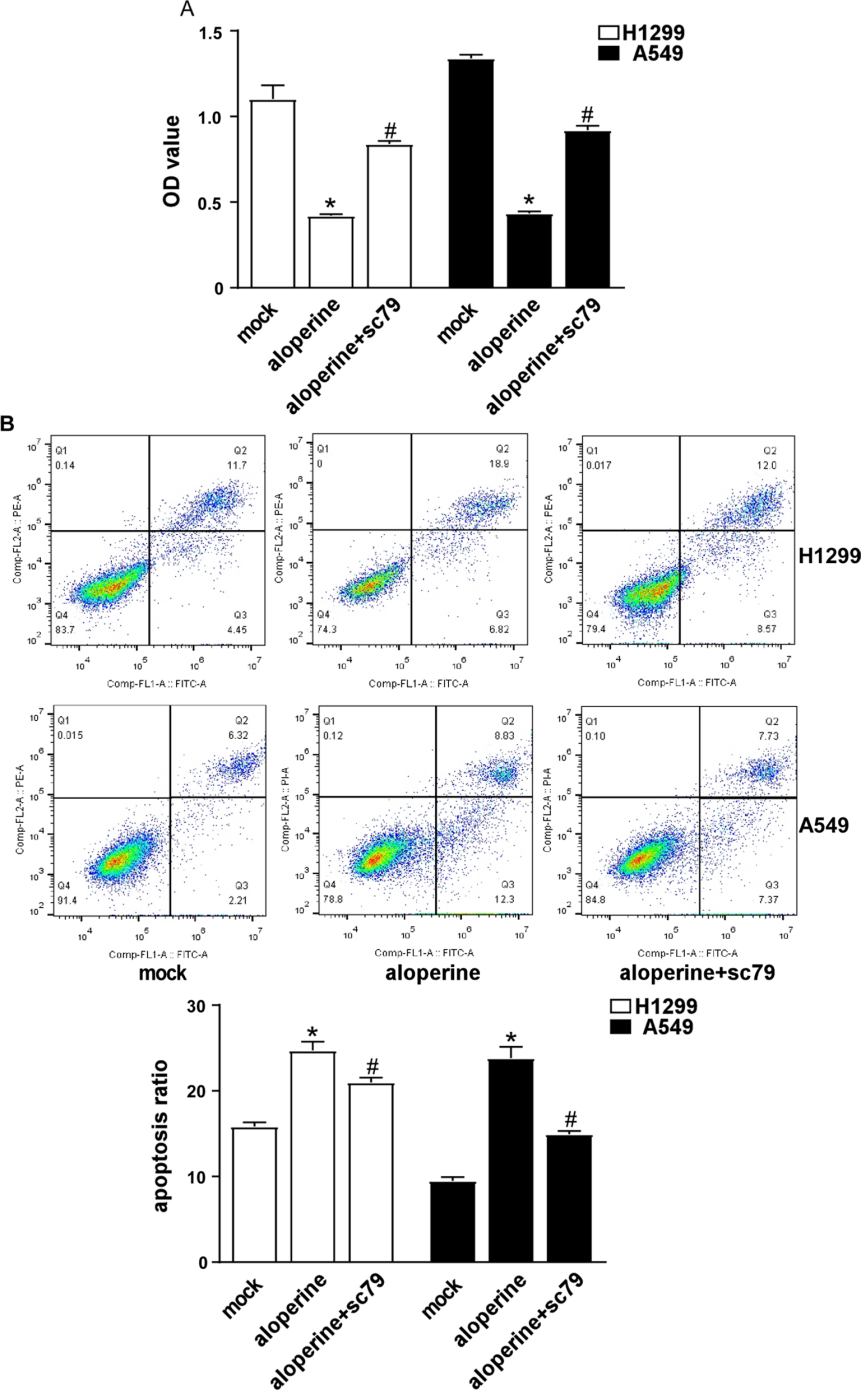
Inflorescence of ALO on the proliferation and apoptosis through PI3K/AKT pathway. **A** H1299 and A549 cells were treated 0.4 mM or 0.3 mM ALO with or without 10uM SC79 for 24 h, respectively. Cells proliferation was detected by CCK8 assay. *P < 0.05, compared to the mock group (solvent ethanol) at 24 h; #P < 0.05, compared to the ALO group (0.4 mM or 0.3 mM). **B** H1299 and A549 cells were treated 0.4 mM or 0.3 mM ALO with or without 10uM SC79 for 24 h, respectively, and then were stained with annexin V/PI, then analyzed by flow cytometry. *P < 0.05, compared to the mock group (solvent ethanol); #P < 0.05, compared to the ALO group (0.4 mM or 0.3 mM). Data are from one experiment representative of three experiments. Two-tailed Student’s test

In order to further verify ALO suppresses cell growth and induces apoptosis through PI3K/AKT pathway, H1299 and A549 cells were treated with 0.4 mM or 0.3 mM of ALO plus 10uM SC79, a p-AKT agonist, for 24 h respectively. The CCK8 assay results showed that SC79 reversed the inhibition of proliferation by ALO for H1299 and A549 cells (Fig. 5A). And the flow cytometry analysis indicated SC79 reversed the induction of apoptosis by ALO (Fig. 5B). These results reveal that ALO could induce apoptosis in NSCLC cells via PI3K/AKT signal pathway.

### ALO attenuates the migration and invasion through PI3K/AKT pathway

**Fig. 6:**
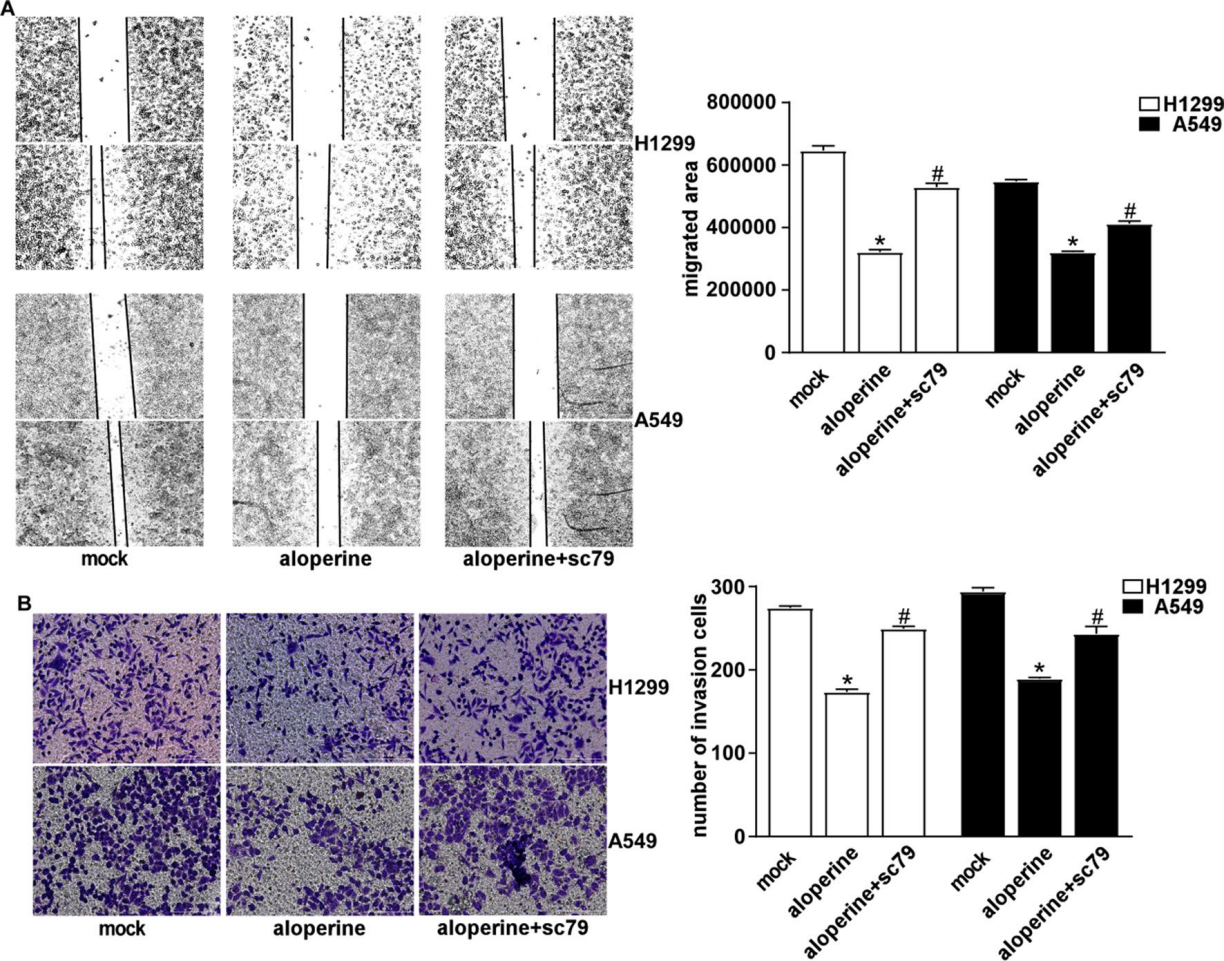
Inflorescence of ALO on the migration and invasion through PI3K/AKT pathway. **A** H1299 and A549 cells were treated 0.4 mM or 0.3 mM ALO with or without 10uM SC79 for 24 h, respectively. The cell migration was detected using wound healing assay. *P < 0.05, compared to the mock group (solvent ethanol) at 24 h; #P < 0.05, compared to the ALO group (0.4 mM or 0.3 mM). **B** H1299 and A549 cells were treated 0.4 mM or 0.3 mM ALO with or without 10uM SC79 for 24 h, respectively, and the cell invasion was detected by transwell assay. *P < 0.05, compared to the mock group (solvent ethanol); #P < 0.05, compared to the ALO group (0.4 mM or 0.3 mM). Data are from one experiment representative of three experiments. Two-tailed Student’s test

In order to further certify ALO treatment also affects the migration and invasion in NSCLC cells through PI3K/AKT pathway, the NSCLC cells were treated with of ALO plus SC79. Wound healing assay showed that SC79 treatment significantly reversed the cell migration inhibited by ALO in H1299 and A549 cells (Fig. 6A). Further, transwell assay indicated that SC79 treatment also reversed the inhibition of H1299 and A549 cells invasion by ALO (Fig. 6B). These datas indicate that ALO could suppress the migration and invasion of NSCLC cells by affecting PI3K/AKT signal pathway.

### ALO suppresses the growth of NSCLC in vivo

**Fig. 7:**
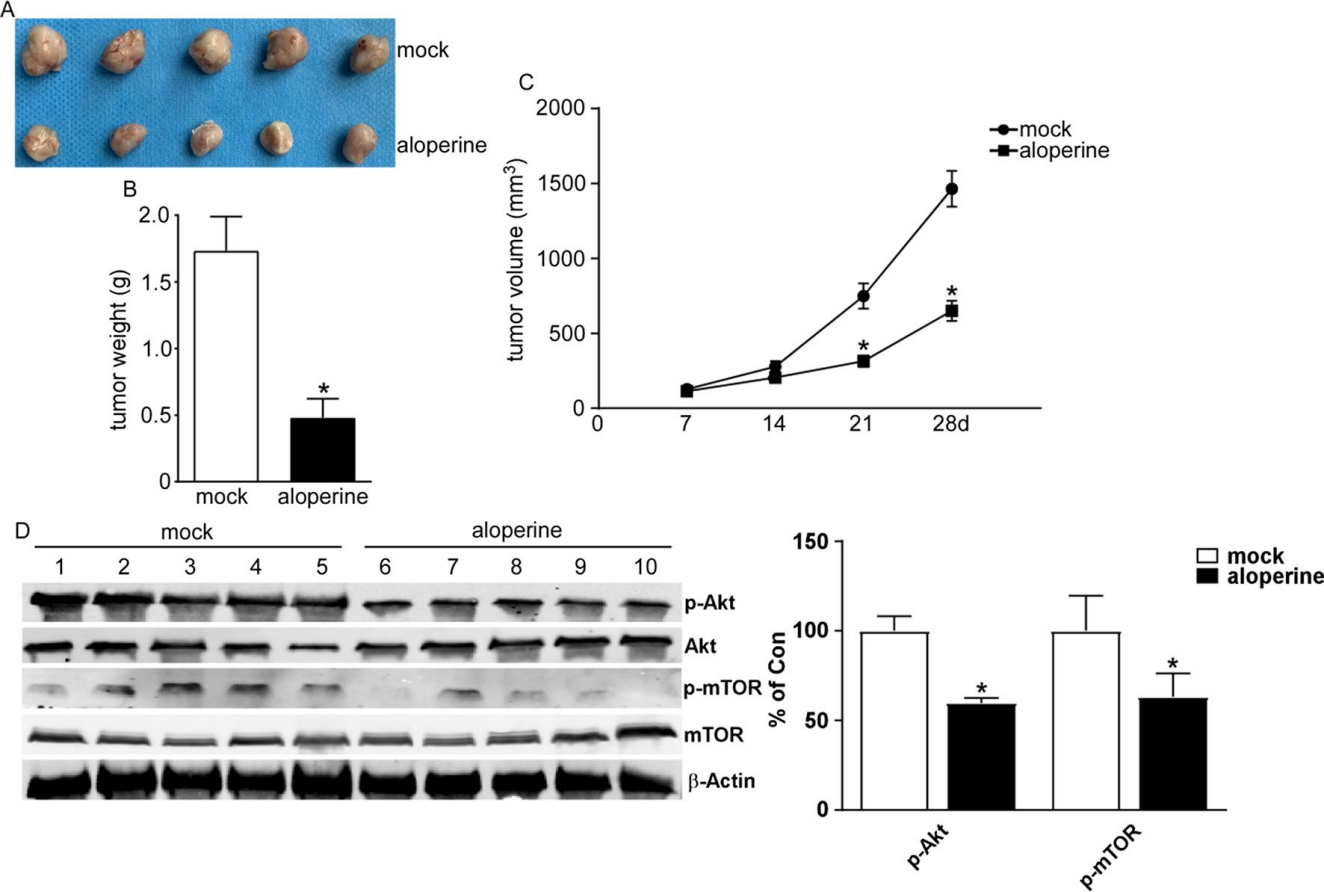
Suppression effect of ALO on tumor growth in vivo*.* **A** Representative tumors images of xenograft mice treated with ALO (30 mg/kg) or normal saline. **B** ALO group displayed a low weight of tumors compared to mock group. *P < 0.05, compared to the mock group (normal saline). **C** The tumors of ALO group showed a more slow growth compared to mock group. *P < 0.05, compared to the mock group (normal saline) at 21d; #P < 0.05, compared to the mock group (normal saline) at 28d. **D** WB images of representative tumors. Small part of xenograft tumor tissue was lysed with RIPA buffer. The tissue lysates were analyzed by Western blotting with the indicated antibodies. *P < 0.05, compared to the mock group (normal saline). Two-tailed Student’s test

To further explore the function of ALO on tumor growth in vivo, A549 cells were used to perform xenograft tumor formation assay. The results revealed that ALO obviously reduced the tumor volume compared to the mock group (Fig. 7A), Moreover, ALO significantly suppressed tumor weight in comparison with mock group (Fig. 7B). Furthermore, the tumors treated with ALO grew more slowly than the mock group (Fig. 7C). These findings showed that ALO inhibited the growth of NSCLC in vivo.

Since ALO induced apoptosis and inhibited invasion of NSCLC cells by inhibiting PI3K/AKT pathway, the effect of tumor growth inhibition by ALO in vivo should be mediated via p-AKT. The p-Akt and p-mTOR of xenograft tumor were determined by western blot. The phosphorylation of Akt and mTOR were also reduced after ALO treatment (Fig. 7D). The results indicated that ALO also inhibited PI3K/AKT pathway in vivo.

## Discussion

Accumulating evidence has indicated that ALO exerts anti-tumor effects on various cancers cells, including leukemia cell lines (HL-60, U937, and K562), esophageal cancer cells (EC109), lung cancer cells (A549), hepatocellular carcinoma cells (HepG2), colon cancer cells (SW480 and HCT116) and osteosarcoma cells (MG-63 and U2OS) [10, 11, 29]. However, the precise molecular mechanism of ALO exerting anti-tumor effects on NSCLC is undefined. In this study, we showed that that ALO inhibited the NSCLC progression in vivo and in vitro. ALO effectively inhibited the proliferation and the clonogenic survival by promoting apotosis in NSCLC. Cell apoptotic effects of ALO were correlated with the activation of caspases family. ALO also suppressed the migration and invasion of NSCLC and this effect was associated with the reduced expression of MMP-2 and MMP-9. Moreover, ALO-induced apoptosis and invasion may be involved in the PI3K/Akt signaling pathway, SC79 as a p-AKT agonist reversed the tumor suppressor function of ALO. SA-49, a new novel aloperine derivative, induced MITF-dependent lysosomal degradation of PD-L1 and suppressed Lewis tumor xenograft growth by activating immune microenvironment in C57BL/6 mice [30]. The current research on SA49 was only limited to antibody-based PD-1/PD-L1 inhibitors for NSCLC, but SA49 maybe became a more reliable and effective anti-tumor compound as the most potential derivative of ALO soon.

Apoptosis refers to a highly regulated physiological process of cell death. Cell apoptosis mainly includes intrinsic pathway (the mitochondrial pathway) and extrinsic pathway (death receptor pathway) [31]. Mitochondria plays a vital role in apoptotic pathway and the mitochondrial dysfunction that occurs during apoptosis [32]. The rupture of mitochondria outer membrane results in the release of cytochrome c into the cytosol. Cytochrome c, together with Apaf-1, forms a heptametrical complex called an apoptosome, resulting in the activation of caspase-9 (initiator caspase) and consequently caspase-3 (executioner caspase). Activated capspase-3 then cleaves PARP, which is known as a marker of apoptosis [33]. Our results showed that ALO activated caspase-9 and caspase-3, as well as PARP, demonstrating that ALO induces apoptosis in NSCLC via the mitochondrial apoptotic pathway. Interestingly, ALO also induces the activation of caspase-9 and caspase-3 in osteosarcoma and hepatocellular carcinoma cells [9, 10]. This indicates that ALO exerts similar anti-tumor functions in different cancer cells.

In addition to apoptosis, necroptosis is an alternative programmed cell death, which is different from apoptosis and that it is regulated by receptor interacting protein kinase-1(RIPK1), RIPK3, and mixed lineage kinase domain-like (MLKL) [34]. Necroptotic pathway may be involved in tumorigenesis [35]. More importantly, Shikonin, is the major constituent of the root of Lithospermum erythrorhizon, could promote RIP1-dependent cell death in cancer cells [36]. Whether ALO can also induce necroptosis in NSCLC will need to be investigated in future.

The distant metastasis is the main cause of morbidity and mortality in patients with cancer [37]. Despite there has been enormous progress in management of NSCLC, survival rates have not improved significantly. More than 79% of all lung cancer patients develop metastases, and the 5-year survival rate of patients with distant metastases is < 5% [38]. Therefore, we conducted wound healing and transwell invasion assay to investigate the influence of ALO on A549 and H1299 cells. Our results indicated that ALO suppressed cell migration and invasion in both cell lines. MMPs are zinc-dependent proteolytic enzymes involved in the degradation of extracellular matrix, such as collagen, fibrinogen and proteoglycan. The expression of MMP-2 and MMP-9 was significantly increased in patients with malignant tumors [39], more importantly, MMP-2 and MMP-9 are essential in in NSCLC invasion and metastasis. Therefore, we explored the expression of MMP-2 and MMP-9 after ALO treatment in A549 and H1299 cells. Our data showed that ALO reduced the expression levels of MMP-2 and MMP-9, suggesting the anti-migrating and anti-invasive roles of ALO in NSCLC.

The underlying mechanisms for ALO-induced anti-tumor effects on lung cancer are well unknown. Muhammed et al. demonstrated that the ALO treatment on NSCLC cells and xenograft model produced anti-proliferative effects, induced apoptosis, and arrested cell cycle at the G1 phase, and the p53/p21 pathway involved in the mechanism [40]. The PI3K/Akt/mTOR signaling pathway has been also implicated in tumor cell proliferation, metastasis and apoptosis [20, 21]. Accumulating evidences suggest that targeting the Akt/mTOR pathway is promising for the treatment of NSCLC [20]. In this study, we found that ALO treatment on NSCLC cells and xenograft model reduced the phosphorylation of Akt and mTOR, but had no effects on the expression of Akt and mTOR. In addition to cancer, the mTOR pathway plays critical roles in the pathogenesis of autoimmune diseases [41] and infectious diseases [42, 43]. Thus, we hypothesized that ALO may be introduced for the treatment of autoimmune and infectious diseases, such as HIV and HBV infection. Interestingly, PP2A was reported to be involved in regulating of Akt activities [44, 45]. PP2A may directly interact with Akt that suppresses PI3K signaling pathway after ALO treatment. Whether PP2A is a direct target of ALO, further studies are needed to investigate.

In summary, our present results suggest that ALO induces apoptosis and invasion in NSCLC through the PI3K/Akt/mTOR signaling pathway. Our study provides a new insight into the use of ALO in the treatment of NSCL.

## Data Availability

The raw materials and data are available from the corresponding author upon reasonable request.
